# The effects of Shenkang suppository on kidney function and gut microbiota in nondialysis patients with chronic kidney disease stages 3–4: A randomized controlled trial

**DOI:** 10.3389/fphar.2025.1621208

**Published:** 2025-07-29

**Authors:** Weiwei Zhang, Xin Zhang, Shuang Xu, Yousuf Abdulkarim Waheed, Yizhou Xu, Shulin Li, Dong Sun

**Affiliations:** ^1^ Department of Nephrology, Affiliated Hospital of Xuzhou Medical University, Xuzhou, China; ^2^ Clinical Research Center for Kidney Disease Xuzhou Medical University, Xuzhou, China; ^3^ Department of Internal Medicine and Diagnostics, Xuzhou Medical University, Xuzhou, China

**Keywords:** chronic kidney disease, shenkang suppository, gut flora, microbiota dysbiosis, traditional Chinese medicine

## Abstract

**Background:**

Chronic kidney disease (CKD) patients have high prevalence and mortality rates; however, current treatment options remain limited. The Shenkang suppository (SKS) is a traditional Chinese medicine used in the clinical management of CKD. Increasing evidence suggests a close relationship between the gut microbiome and CKD. We aimed to investigate the impact of SKS on kidney function and the gut microbiota in CKD stage 3–4 patients.

**Methods:**

It’s a single-center randomized controlled trial (n = 80). CKD stage 3–4 patients were randomly assigned at a 1:1 ratio to either the control group (n = 40) or the SKS group (n = 40) and followed for 8 weeks. Baseline data, clinical indicators, kidney markers, and stool samples were obtained from participants before and after treatment. The bacterial DNA was isolated from fecal samples and analyzed via high-throughput 16S rRNA sequencing.

**Results:**

A total of 34 patients in the SKS group and 36 in the control group completed the study. Kidney function in the control group significantly worsened after 8 weeks (*P < 0.05*), whereas SKS had a positive effect on slowing the progression of kidney function decline. After 8 weeks of SKS intervention, the beneficial bacteria Coriobacteriaceae and Ruminococcaceae-*Ruminococcus* proliferated dramatically, whereas the abundance of the harmful bacteria Veillonellaceae decreased. Linear discriminant analysis (LDA) revealed that SKS treatment increased the proportion of *Streptococcus* and decreased the proportions of Planctomycetes, Spirochaetes, Rhodocyclales, Actinomycetaceae, Veillonellaceae, and Dechloromonas. These changes were not observed in the control group. Correlation analysis revealed a negative association between *Bifidobacterium*, *Blautia*, Enterococcaceae_*Enterococcus*, and clinical kidney function indicators, whereas *Parabacteroides*, *Acinetobacter*, *Sutterella*, *Oscillospira*, and *Alistipes* were positively correlated with kidney function.

**Conclusion:**

SKS could delay the progression of kidney function in CKD stage 3–4 patients, possibly by modulating the dysbiosis of the gut microbiota. The close associations between certain gut microbiota and clinical kidney function indicators suggest that the gut microbiota could serve as a new therapeutic target for CKD.

**Clinical Trial Registration:**

https://www.chictr.org.cn/, Chinese Clinical Trial Registry Identifier: ChiCTR2200058493.

## Introduction

CKD is described as a chronic and permanent impairment of kidney function and structure and is a significant public health concern. CKD has affected approximately 13.4% of the global population (Hill et al., 2016). CKD patients usually require dialysis or kidney transplantation to survive when progressed to end-stage kidney disease (ESKD). Early interventions may help reduce the incidence of ESKD (National Kidney Foundation, 2012). However, current treatment strategies to attenuate the progression of renal dysfunction are limited. Thus, identifying novel therapeutic targets is crucial for attenuating the progression of CKD and improving the prognosis of this population.

The gut microbiota, which comprises approximately 100 trillion microorganisms, serves as the largest symbiotic ecosystem of the human body, including bacteria, fungi, and viruses (Lin and Zhang, 2017). These microbes are involved in various metabolic activities, including the synthesis of vitamins, secondary bile acids, and choline and the production of short-chain fatty acids (SCFAs) (Ikeda et al., 2022). SCFAs are metabolic byproducts of gut microbes and are produced through the anaerobic fermentation of dietary fibers. Research has reported that they offer nephroprotective benefits, such as anti-inflammatory, anti-atherosclerotic, and antioxidant properties (Corte-Iglesias et al., 2024). Additionally, the gut microbiota plays an important role in maintaining immunological function and preserving gut barrier integrity (Rackaityte and Lynch, 2018). Several animal and clinical studies revealed that gut microbiota dysbiosis is linked to various diseases, including cardiovascular disease (CVD), obesity, diabetes, inflammatory bowel disease, nonalcoholic fatty liver disease, and mental health disorders (Young, 2017; Tang et al., 2017; Bajzer and Seeley, 2006; Ma et al., 2019; Weingarden and Vaughn, 2017; Fang et al., 2022; Shoubridge et al., 2022).

There is growing evidence suggesting that the gut microbiota is connected with the progression of CKD and its complications (Lohia et al., 2022). CKD patients are particularly susceptible to gut dysbiosis and intestinal barrier dysfunction (Sabatino et al., 2015). These factors include reduced dietary fiber intake, impaired gut motility, and impaired protein assimilation (Bammens et al., 2003). Gut dysbiosis and disruption of intestinal barrier function are key factors in the interaction between the gut microbiota and CD and have been the focus of previous studies. Dysbiosis triggers systemic chronic inflammation and the production of uremic toxins (Hou et al., 2022; Huang et al., 2017). These toxins enter the bloodstream through the weakened intestinal barrier, accumulate in circulation, and are challenging to remove by dialysis (Gryp et al., 2017). The accumulation of uremic toxins activates inflammation, oxidative stress, and fibrosis pathways, increasing the incidence and mortality of CVD and other CKD-related complications (Felizardo et al., 2019). Studies indicate that uremic toxins can enhance NADPH oxidase activity and reactive oxygen species (ROS) production (Watanabe et al., 2013). Wang et alhypothesized that dysbiosis in CKD may disturb toxic and prooxidant metabolitesdirectly or indirectly by regulating vitamin and cofactormetabolism, such as hydroquinone from ubiquinone andother terpenoid-quinone biosynthesis pathways (Wang et al., 2023). This accelerates renal function decline and creates a vicious cycle. Thus, modulation of the gut microbiota may be a new target for slowing CKD progression.

Traditional Chinese medicine (TCM) is considered a potential therapeutic option for CKD because of its definite efficacy, broad application range, and minimal side effects. TCM has demonstrated beneficial effects in controlling proteinuria, protecting renal function, and improving clinical symptoms in CKD patients (Chen et al., 2013). Studies have suggested that TCM may serve as an important exogenous modulator in the interaction between the gut microbiome and CKD (Sun et al., 2022). Research has shown that TCM can alter the diversity and composition of the gut microbiota, thereby alleviating associated diseases (Xu et al., 2017).

SKS is a compound TCM formulation that mainly includes the herbs Rheum (Rhubarb), Astragalus, Salvia miltiorrhiza, and Carthamus tinctorius, which are all commonly used in Chinese herbal medicine. Increasing evidence suggests that Rhubarb can affect the gut microbiota (Luo et al., 2019) and improve intestinal mucosal barrier function (Wang et al., 2017). Zeng et al. demonstrated that rhubarb extract could regulate the gut microbiome in CKD rats, reduce uremic toxin production, and alleviate kidney damage (Zeng et al., 2016). Moreover, Han et al. reported that combining Astragalus and Salvia miltiorrhiza can lessen kidney injury and increase beneficial gut bacteria (Han et al., 2019). Additionally, SKS, which is administered rectally, can directly stimulate the intestinal mucosa, cause intestinal congestion, and increase the permeability of the mucosal capillaries. The sennoside and anthraquinone components in Rhubarb promote intestinal motility and bowel movement, thereby assisting in the excretion of uremic toxins via the intestine (Hoibian et al., 2018).

However, the precise impact of SKS on the gut microbiota in CKD patients remains unclear. Thus, our study aimed to evaluate whether the traditional herbal formulation SKS can improve renal function in patients with CKD stages 3–4 by modulating the gut microbiota and to identify potential therapeutic targets for CKD treatment.

## Methods and analysis

### Study design and randomization

We conducted a single-center, randomized controlled trial at the Department of Nephrology, The Affiliated Hospital of Xuzhou Medical University, from August 2022 to May 2024. A total of 80 participants with CKD stages 3–4 were enrolled in the current study. Upon enrollment, all patients received dietary recommendations based on the 2020KDOQI Clinical Practice Guideline for Nutrition in CKD and were encouraged to maintain stable dietary intake (Ikizler et al., 2020). The enrolled participants were randomly allocated into two groups at a 1:1 ratio via therandom number table method: the control group received conventional renal conservation treatment (high-quality protein, low salt, low fat, low phosphorus diet, control of blood pressure, blood glucose, etc.), whereas the SKS group received conventional treatment combined with SKS. For the initial 2 weeks, patients in the SKS group were administered two suppositories, one in the morning and one in the evening, with gradual dose escalation on the basis of tolerance. After 2 weeks, the dose was increased to five suppositories per day, taken in three doses (morning, noon, and evening), with two suppositories administered rectally at bedtime. The total treatment duration was 8 weeks. Any adverse events were recorded during the study. Each investigator conducted the study in compliance with the local or regional regulatory requirements and with the ethical standards of the hospital. This study was approved by the Ethics Committee of the Affiliated Hospital of Xuzhou Medical University (XYFY2021-KL228-01) and registered with the Chinese Clinical Trial Registry (ChiCTR2200058493). Informed consent was obtained from all participants before enrollment.

### Sample size calculation

Conventional biostatistical criteria suggest that a sample of 80 patients (40 patients in each group) is ideal to achieve adequate power (≥80%) for sample size justification (Daniël, 2022). This applies to kidney or gut outcomes measured after 8 weeks (with α = 0.05; two-tailed tests). For moderate effect sizes (d = 0.5), a total of 126 subjects is needed. Deciding upon the condition of eGFR changes assumes a clinically meaningful difference towards the 5 mL/min/1.73 m^2^, and a standard deviation of 8 mL/min/1.73 m^2^ (resulting in a standardized effect size of d = 0.625), having 40 patients per group would provide a power of only 79%, which is marginally acceptable but below the standard threshold. Biologically, the effectiveness of Shenkang has been reinforced by strong network pharmacology evidence, where similar sample sizes *in vitro* studies (n = 35–45 per group) have detected significant reductions in creatinine and oxidative biomarkers (Liu et al., 2023). Therefore, the sample of 80 patients could be underpowered unless there is strong earlier evidence of large treatment effects or low variability of outcomes; however, future randomized studies should strive to recruit larger numbers of participants so proportionately larger effect sizes can be demonstrated.

### Participants

The inclusion criteria were as follows: (1) aged 18–70 years at the time of enrollment; (2) CKD stage 3–4 with an estimated glomerular filtration rate (eGFR) between ≥15 and <60 mL/min/1.73 m^2^ in accordance with the Kidney Disease: Improving Global Outcomes (KDIGO) guidelines (Kidney Disease: Improving Global Outcomes CKD Work Group, 2024). The exclusion criteria were as follows: (1) history of antibiotic, probiotic, prebiotic, or synbiotic use within the past 4 weeks; (2) active inflammatory disease within the last 2 weeks; (3) known gastrointestinal or systemic diseases that affect the gut composition; (4) severe perianal or rectal diseases; (5) pregnancy or breastfeeding, and those with allergies to any ingredients in the SKS. The discontinuation and withdrawal criteria were as follows: (1) poor adherence to the prescribed treatment regimen, (2) voluntary withdrawal by the participant, and (3) severe adverse reactions.

### Shenkang suppository

The Shenkang suppository (3 g × 7 suppositories, Xi’an Century Shengkang Pharmaceutical Co., Ltd.) was approved by the National Medical Products Administration (Z20050482). According to the guidelines, patients were informed that the medication may induce a sensation of the need to defecate or an increase in bowel movements, which is a normal effect of the drug. Patients were also advised that some individuals may experience side effects such as perianal burning, abdominal pain, or diarrhea after using the medication.

### Outcome assessments

We collected clinical information, including baseline characteristics such as age, sex, body mass index (BMI), and laboratory markers such as blood pressure, serum albumin, total cholesterol, blood glucose, hemoglobin, serum potassium, and phosphate levels. Blood samples, including measurements of uric acid (UA), blood urea nitrogen (BUN), cystatin C (Cys-C), and serum creatinine (SCr), were collected before and after intervention to assess renal function. The eGFRs were calculated according to the modified 2006 IMDS-MDRD formula, which is based on serum creatinine levels, sex, and age at the time of enrollment (Levey et al., 2006).

### Fecal sample collection and genomic DNA extraction

Each participant provided approximately 1 g of fresh fecal sample in a 5 mL sterile tube at the beginning of the study and after 8 weeks of intervention. The samples were stored at −80°C within 30 min of collection. Once sample collection was complete, all samples were retrieved from the freezer, and an appropriate amount (0.2–0.5 g) was extracted for DNA isolation via the OMEGA Soil DNA Kit (D5635–02) (Omega Bio-Tek, Norcross, GA, United States). The 16S rRNA V3-V4 region was subsequently amplified via PCR via specific primers.

### 16S rRNA gene sequencing data analysis

The 16S V3-V4 variable region extracted from human fecal samples was sequenced on the NovaSeq platform. We used the QIIME2 platform,and the microbiome’s biological information was analyzed according to the official guidelines. The raw sequence data were decoded with the Demux plugin, and the primers were trimmed via Cutadapt. Data processing, including quality filtering, denoising, merging, and chimera removal, was performed with DADA2. The sequences were merged at 100% similarity, generating ASVs and an abundance table. The sequences were denoised and classified using Greengenes as the reference database.

### Statistical analysis

Continuous variables with a normal distribution are expressed as the means ± standard deviations, whereas nonnormally distributed variables are presented as interquartile ranges. Categorical data are shown as percentages. For normally distributed data, intergroup comparisons were made via the independent samples ttest, and intragroup comparisons were made via the paired samples ttest. For nonnormally distributed data, the Mann‒Whitney U test was used for intergroup comparisons, and the Wilcoxon signed-rank test was used for intragroup comparisons. Spearman’s rank correlation was used to analyze the relationships between the microbiota and clinical indicators. All analyses were performed via SPSS version 25, with *P < 0.05* considered statistically significant.

## Results

### Baseline characteristics of the participants

**FIGURE 1:**
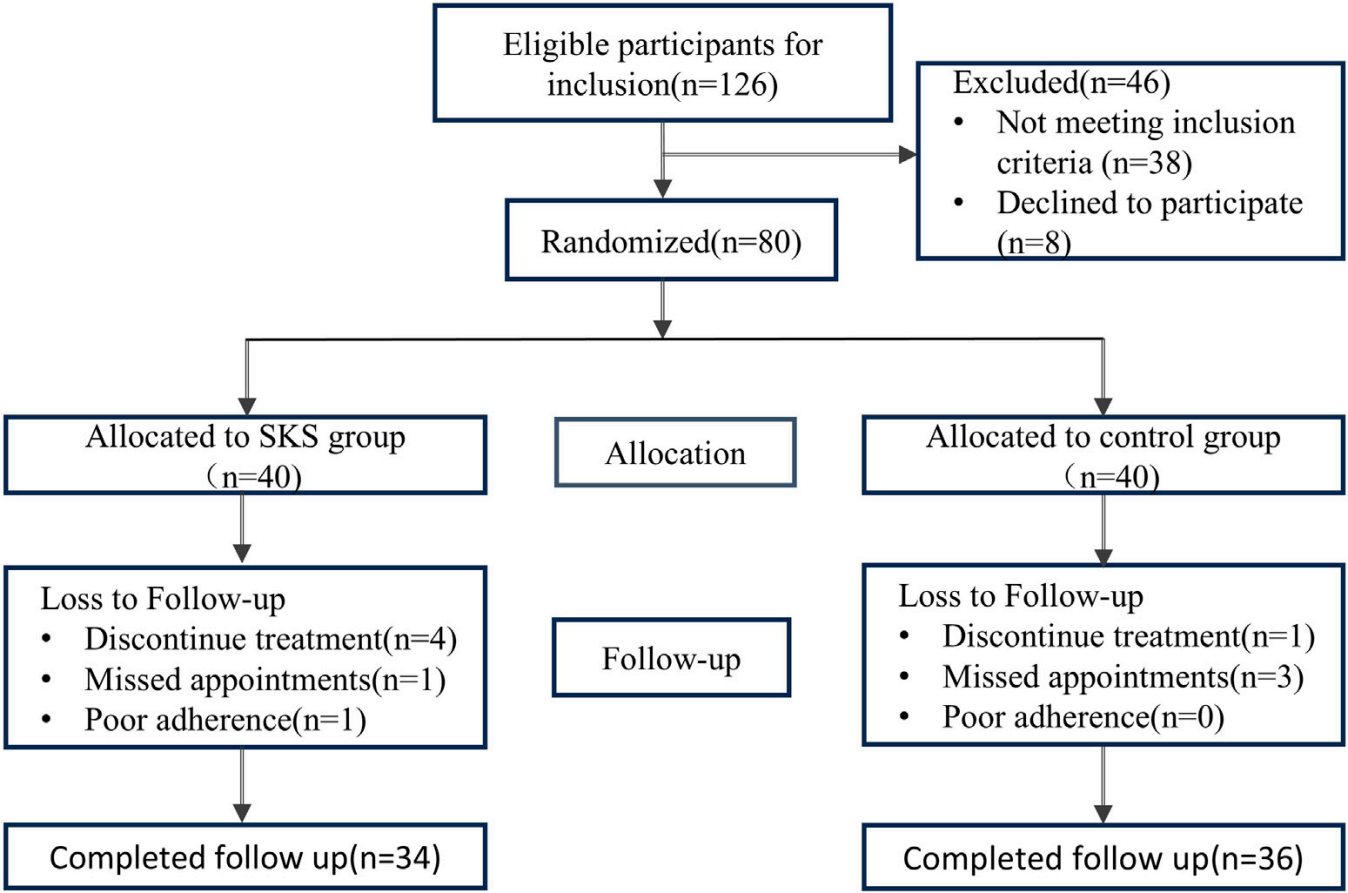
Flowchart of the 8 weeks randomized controlled trial. Flowchart of the 8 weeks randomized controlled trial (126) Patients were assed for eligibility, 46 patients were exclude for different reasons, 80 patients included as an initial step, randomized in a 1:1 ratio to either SKS group or control group. Patients with missing appointments or poor compliance were excluded from the trial.

From August 2022 to May 2024, 80 participants were randomized (SKS group 40; control group 40). Among the 80 patients, 70 completed the trial, with a mean age of 51.56 ± 8.74 years and 48.72 ± 12.63 years in the SKS and control groups, respectively (*P < 0.234*). Five participants (7.14%) who missed their appointments were excluded from the trial, and 5 (7.14%) who discontinued the treatment were also excluded. Onepatient (1.42%) who had poor compliance was also excluded. For more details, please refer to (Figure 1). At baseline, laboratory markers were not significantly different between the two groups (*P > 0.05*), except for potassium (K), which was significantly different between the two groups (4.52 ± 0.47 mmol/L vs. 4.26 ± 0.60 mmol/L in the SKS and control groups, respectively; *P < 0.051*). The mean eGFRs were 33.64 ± 12.56 mL/min and31.98 ± 12.65 mL/min in the SKS and control groups, respectively (*P < 0.581*). The participants were evenly distributed between the two groups. For more details, please refer to (Table 1) for the baseline characteristics of both groups.

**TABLE 1:** Baseline characteristics of the study population.

Characteristics	SKS group	Control group	t/χ^2^	*P value*
Number of Patients	N = 34	N = 36		
Demographical				
Gender				
Male patients (%)	20 (59)	16 (44)		
Female patients (%)	14 (41)	20 (56)		
Age, years	51.56 ± 8.74	48.72 ± 12.63	−1.10	0.276
BMI, kg/m2	25.40 ± 4.38	24.22 ± 3.88	−1.20	0.234
SBP, mmHg	132.59 ± 14.51	139.36 ± 15.91	1.86	0.068
DBP, mmHg	84.91 ± 11.96	84.39 ± 13.47	−0.17	0.864
Laboratory				
ALB, g/L	41.05 ± 4.53	38.21 ± 6.71	−1.78	0.076
TC, mmol/L	4.91 ± 1.16	4.98 ± 1.29	0.23	0.822
GLU, mmol/L	5.22 ± 1.18	5.29 ± 1.70	0.21	0.833
Hgb, g/L	120.79 ± 19.12	111.69 ± 22.03	−1.84	0.070
K, mmol/L	4.52 ± 0.47	4.26 ± 0.60	−1.98	0.051
P, mmol/L	1.23 ± 0.21	1.22 ± 0.24	−0.12	0.905
Scr, mg/dL	2.28 ± 0.93	2.33 ± 0.89	0.20	0.843
^a^eGFR, mL/min/1.73 m2	33.64 ± 12.56	31.98 ± 12.65	−0.55	0.581
Cys-C, mg/L	2.25 ± 0.59	2.25 ± 0.57	0.01	0.996
BUN, mmol/L	13.26 ± 4.67	12.46 ± 4.85	−0.70	0.484
UA, mg/dL	6.58 ± 1.82	6.30 ± 1.59	−0.67	0.502
Medications				
Antiplatelet agent (%)	28 (82)	26 (72)	1.02	0.314
Diuretics (%)	11 (32)	14 (39)	0.32	0.570
ACEI/ARB (%)	12 (35)	13 (36)	0.01	0.943
β-blocker (%)	8 (24)	7 (19)	0.17	0.677
Insulin (%)	3 (9)	2 (6)	0.28	0.595

Variables are expressed as a mean ± SD, or expressed as (Percentage %). No significant differences were observed between the different analyzed variables at baseline except of potassium K. Student’s t-test was utilized to compare the normal distributed data.

^a^ eGFR (ml/min per 1.73 m2) was calculated according to the modified 2006 IMDS-MDRD, formula which is based on serum creatinine levels, gender, and age.

P *< 0.05* means statistically difference.

Abbreviations: SKS, shenkang suppository; SCr, serum creatinine; eGFR, estimated glomerular filtration rate; Cys-C, cystatin C; BUN, blood urea nitrogen; UA: uric acid; BMI, body mass index; SBP, systolic blood pressure; DBP, diastolic blood pressure; ALB, serum albumin; TC, total cholesterol; GLU, glucose; Hgb:hemoglobin; K, serum potassium; P, serumphosphate; ACEI/ARB, Angiotensin-Converting Enzyme Inhibitor/Angiotensin Receptor Blocker.

### Effect of SKS on the progression of kidney disease

We compared the changes in renal function before and after treatment between the two groups. The Q‒Q plot revealed that the data were approximately normally distributed; therefore, we utilized Student’s t-test to compare the two groups. Our results revealed no significant difference in renal function between the groups before treatment (*P < 0.05*) (see Table 2).

**TABLE 2:** Changes of kidney function in both group after 8-week of treatment.

Variable	SKS group	Control group	t [95%CI]	*P value*
No. of patients	N = 34	N = 36		
SCr,mean(SD), mg/dL				
Baseline	2.28 ± 0.93	2.33 ± 0.89	0.198 [−0.39 to 0.48]	0.843
8 weeks	2.13 ± 0.86	2.79 ± 1.28	2.540 [0.14 to 1.18]	0.014
Cys-C,mean(SD), mg/L				
Baseline	2.25 ± 0.59	2.25 ± 0.57	0.005 [−0.27 to 0.28]	0.996
8 weeks	2.17 ± 0.62	2.51 ± 0.69	2.170 [0.03 to 0.65]	0.034
^a^eGFR,mean(SD),mL/min/1.73 m2				
Baseline	33.64 ± 12.56	31.98 ± 12.65	−0.554 [−7.69 to 4.35]	0.581
8 weeks	36.89 ± 14.52	28.14 ± 13.46	−2.615 [−15.42 to −2.07]	0.011
BUN,mean(SD),mmol/L				
Baseline	13.26 ± 4.67	12.46 ± 4.85	−0.704 [−3.07 to 1.47]	0.484
8 weeks	12.65 ± 5.51	14.02 ± 5.55	1.035 [−1.27 to 4.01]	0.304
UA, mean (SD), mg/dL				
Baseline	6.58 ± 1.82	6.30 ± 1.59	−0.674 [−1.09 to 0.54]	0.502
8 weeks	6.95 ± 1.87	6.43 ± 1.51	−1.280 [−1.33 to 0.29]	0.205

Variables are expressed as a mean ± SD., no significant differences were observed between the different analyzed variables at baseline.

^a^ eGFR (ml/min per 1.73 m2) was calculated according to the modified 2006 IMDS-MDRD, formula which is based on serum creatinine levels, gender, and age.

P *< 0.05* means statistically difference.

Abbreviations: SKS, shenkang suppository; SCr, serum creatinine; eGFR, estimated glomerular filtration rate; Cys-C, cystatin C; BUN, blood urea nitrogen; UA: uric acid.

After 8 weeks of treatment, a significant difference in the SCr level was observed between the two groups (2.13 ± 0.86 in the SKS group vs. 2.79 ± 1.28 in the control group, *P < 0.014;* Table 2). Similarly, the eGFR also significantly differed after 8 weeks of treatment (36.89 ± 14.52 in the SKS group vs. 28.14 ± 13.46 in the control group, *P < 0.011;* Table 2). Additionally, we observed a significant difference in Cys-C levels after 8 weeks of treatment (2.17 ± 0.62 in the SKS group vs. 2.51 ± 0.69 in the control group, *P < 0.034;* Table 2). However, no significant differences were found in the BUN and UA levels after 8 weeks of treatment (*P < 0.304* and P < 0.205, respectively) (see Table 2).

These findings suggest that SKS could have the potential to slow the progression of renal function decline in CKD stage 3–4 patients.

### Effect of treatments on kidney function within each group

To explore the effects of SKS on renal function in CKD patients in greater detail within each group, we also compared the renal parameters before and after 8 weeks of treatment within the groups.

After 8 weeks of treatment, the eGFR in the SKS group increased compared with that at baseline (33.64 ± 12.56 at baseline vs. 36.89 ± 14.52 after 8 weeks, *P < 0.03;* Table3). Moreover, the eGFR in the control group decreased from baseline (31.98 ± 12.65 at baseline vs. 28.14 ± 13.46 after 8 weeks, *P < 0.002;* Table3). SCr also significantly differed between the SKS group and the control group before and after 8 weeks of treatment.

**TABLE 3:** Changes in kidney function biomarkers before and after 8-week of treatment within each group.

Variables	SKS group (n = 34)				Control group (n = 36)			
	Baseline	8 weeks	t	*P value*	Baseline	8 weeks	t	*P value*
SCr, mg/dL	2.28 ± 0.93	2.13 ± 0.86	2.625	0.013	2.33 ± 0.89	2.79 ± 1.28	−4.784	<0.001
Cys-C, mg/L	2.25 ± 0.59	2.13 ± 0.86	1.293	0.205	2.25 ± 0.57	2.51 ± 0.69	−4.055	<0.001
eGFR, ml/min/1.73m2	33.64 ± 12.56	36.89 ± 14.52	−2.268	0.030	31.98 ± 12.65	28.14 ± 13.46	3.259	0.002
BUN, mmol/L	13.26 ± 4.67	12.65 ± 5.51	0.776	0.443	12.46 ± 4.85	14.02 ± 5.55	−2.826	0.008
UA, mg/dL	6.58 ± 1.82	6.43 ± 1.51	−0.944	0.352	6.30 ± 1.59	6.43 ± 1.51	−0.462	0.647

Measurement data are given as mean ± standard deviation, the end point of the study was compared with that before treatment.

Paired samples t-test was used to compare the variables, *P < 0.05* is considered a statistical significant.

Abbreviations: SKS, shenkang suppository; SCr, serum creatinine; eGFR, estimated glomerular filtration rate; Cys-C, cystatin C; BUN, blood urea nitrogen; UA: uric acid.

Similarly, the BUN level decreased in the SKS group after 8 weeks of treatment (13.26 ± 4.67 at baseline vs. 12.65 ± 5.51 after 8 weeks, *P < 0.443*; Table3). Although it was not statistically significant, it slowed the progression of CKD. The control group presented an increase in BUN after 8 weeks of treatment (12.46 ± 4.85 at baseline vs. 14.02 ± 5.55 after 8 weeks, *P < 0.008*; Table3).

Additionally, Cys-C was not significantly different within the SKS group after treatment (2.25 ± 0.59 at baseline vs. 2.13 ± 0.86 after 8 weeks, *P < 0.205;* Table 3). On the other hand, the control group presented a significant increase in Cys-C levels from baseline (2.25 ± 0.57 at baseline vs.2.51 ± 0.69 after 8 weeks, *P < 0.001*; Table3).

No significant changes were observed in the serum UA levels in either group. Overall, our findings indicate that SKS may have the potential to slow the progression of stage 3–4 CKD patients.

### Effects of SKS on the gut microbiota composition

**FIGURE 2:**
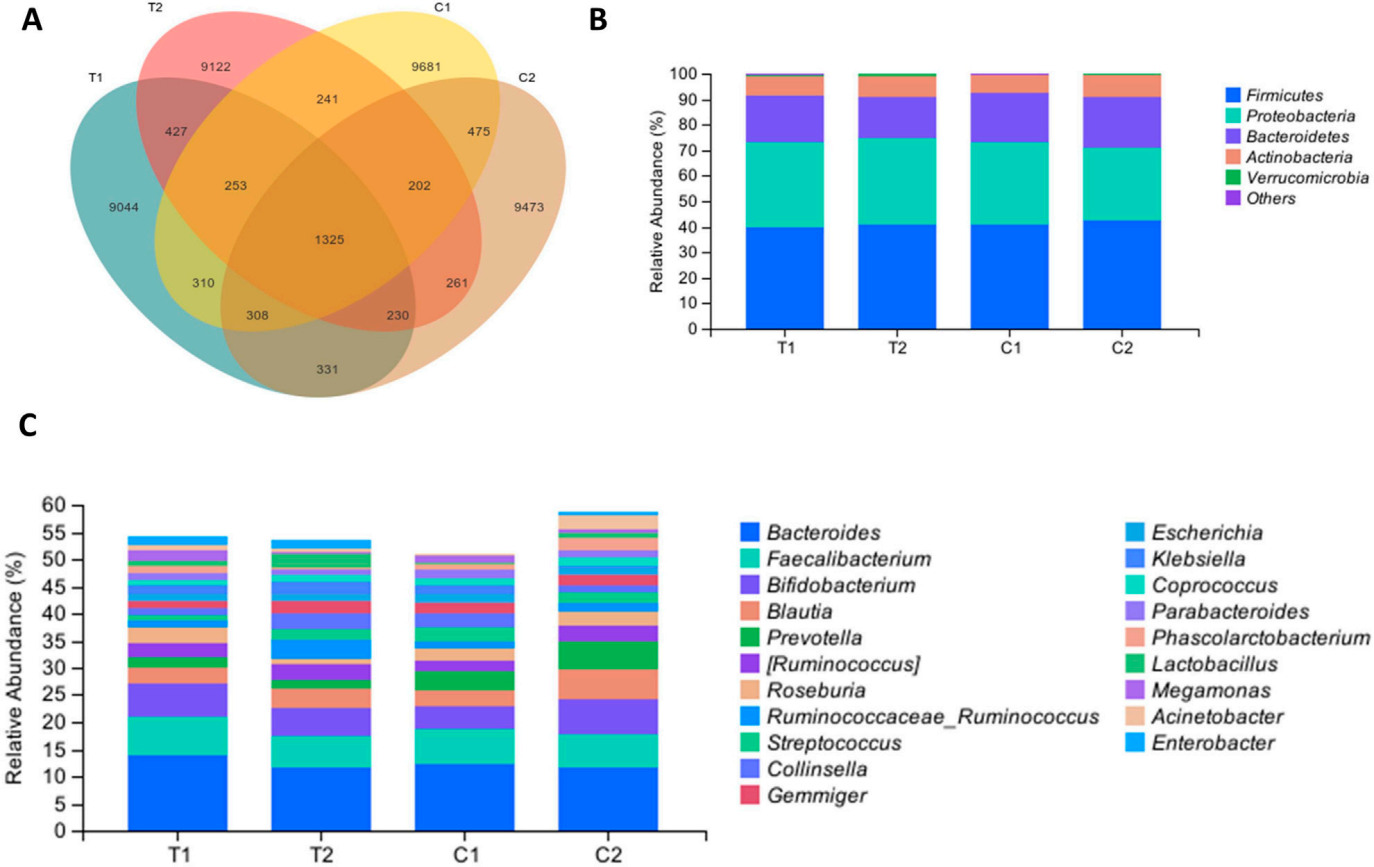
Effects of SKS on the gut microbiota composition. **(A)**:Venn diagramdepicting the unique and shared ASVs inthe control and YSHS group at baseline (C1, T1) and 8-week follow-up (C2, T2). **(B)**:Relative abundance of the gut microbiota in chronic kidneydisease patients in the control and SKS group at baseline (C1, T1) and 8-week follow-up (C2, T2) at the phylum level. **(C)** Relative abundance of the gut microbiota in chronic kidneydisease patients in the control and SKS group at baseline (C1, T1) and 8-week follow-up (C2, T2) at the genus level.

**FIGURE 3:**
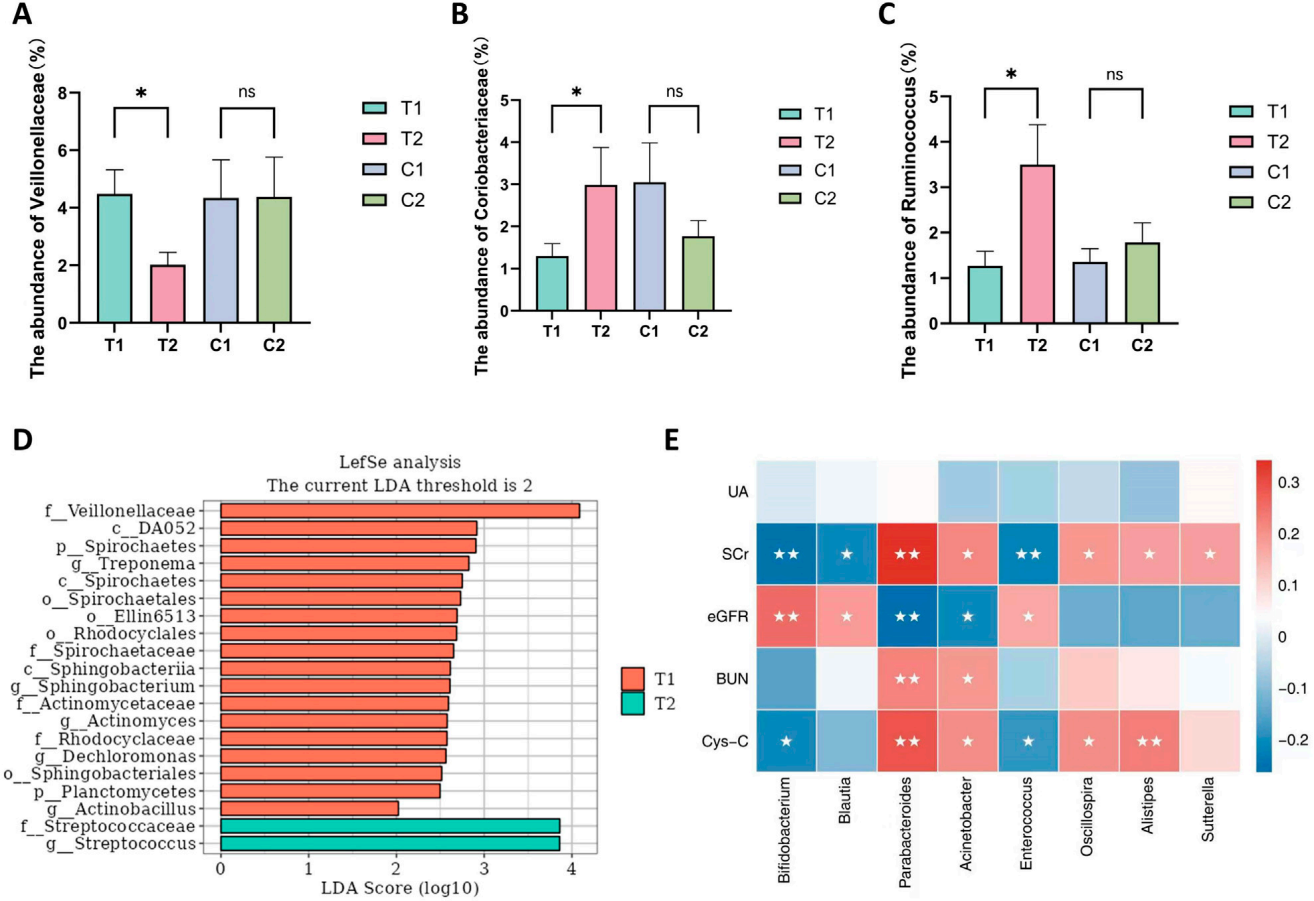
The significantly altered microbiota in the SKS group. and the correlation between microbiota and kidney function. **(A–C)**: The abundance of Veillonellaceae、Coriobacteriaceae、Ruminococcaceae-Ruminococcusbetween patientsin the control and SKS group at baseline (C1, T1) and at 8-week follow-up (C2, T2). **(D)** Histogram of the LDA scores computedfor differentially abundant bacterial taxa between baseline (T1) and 8-week follow-up (T2) in the SKS group. **(E)**:Heatmaps showing correlations between differentially abundant microbiotagenera and clinical parameters in chronic kidney disease patients treated with SKS. Note:nsmeans no statistically difference; *means P < 0.05; **means P < 0.01.

Both groups provided 140 fecal samples in total, with an average of 521 amplicon sequence variants (ASVs) obtained per sample. Figure 2A shows a Venn diagram depicting the unique and shared ASVs between groups. Statistical analysis of the relative abundance of the feature sequences revealed varying degrees of change at each taxonomic level. As shown in Figure 2B, at the phylum level, Firmicutes was the most abundant phylum, with relative abundances of 39.79% and 40.92% in the two groups at baseline, followed by Proteobacteria, Bacteroidetes, and Actinobacteria, which together accounted for more than 90% of the microbiota. Compared with the control group, the SKS group presented a reduction in Bacteroidetes after treatment and an increase in the Firmicutes/Bacteroidetes (F/B) ratio, although these differences were not statistically significant. At the family level, the SKS group presented a significant decrease in Veillonellaceae (*P < 0.036*) and a significant increase in Coriobacteriaceae (*P* < *0.048*) (Figures 3A,B). (Figure 2C) shows the abundance of the gut microbiota at the genus level, revealing a significant increase in the relative abundance of Ruminococcaceae-*Ruminococcus* in the SKS group (*P* < *0.023*) (Figure 3C). These results confirm the impact of SKS on the composition of the gut microbiota.

To further assess the differences in the abundance of identified ASVs between the two groups, we performed an LDA effect size (LEfSe) analysis, setting the LDA threshold at >2 and the Pvalue at <0.05. The LEfSe analysis results revealed several differential biomarkers before and after treatment in the SKS group. In the T1 group, 11 gut microbiota taxa were enriched, including p_Planctomycetes, p_Spirochaetes, o_Rhodocyclales, f_Actinomycetaceae, f_Veillonellaceae, and *g_Dechloromonas*. The T2 group was enriched in f_Streptococcaceae (Figure 3D).

### Effects of SKS on gut microbial diversity

**FIGURE 4:**
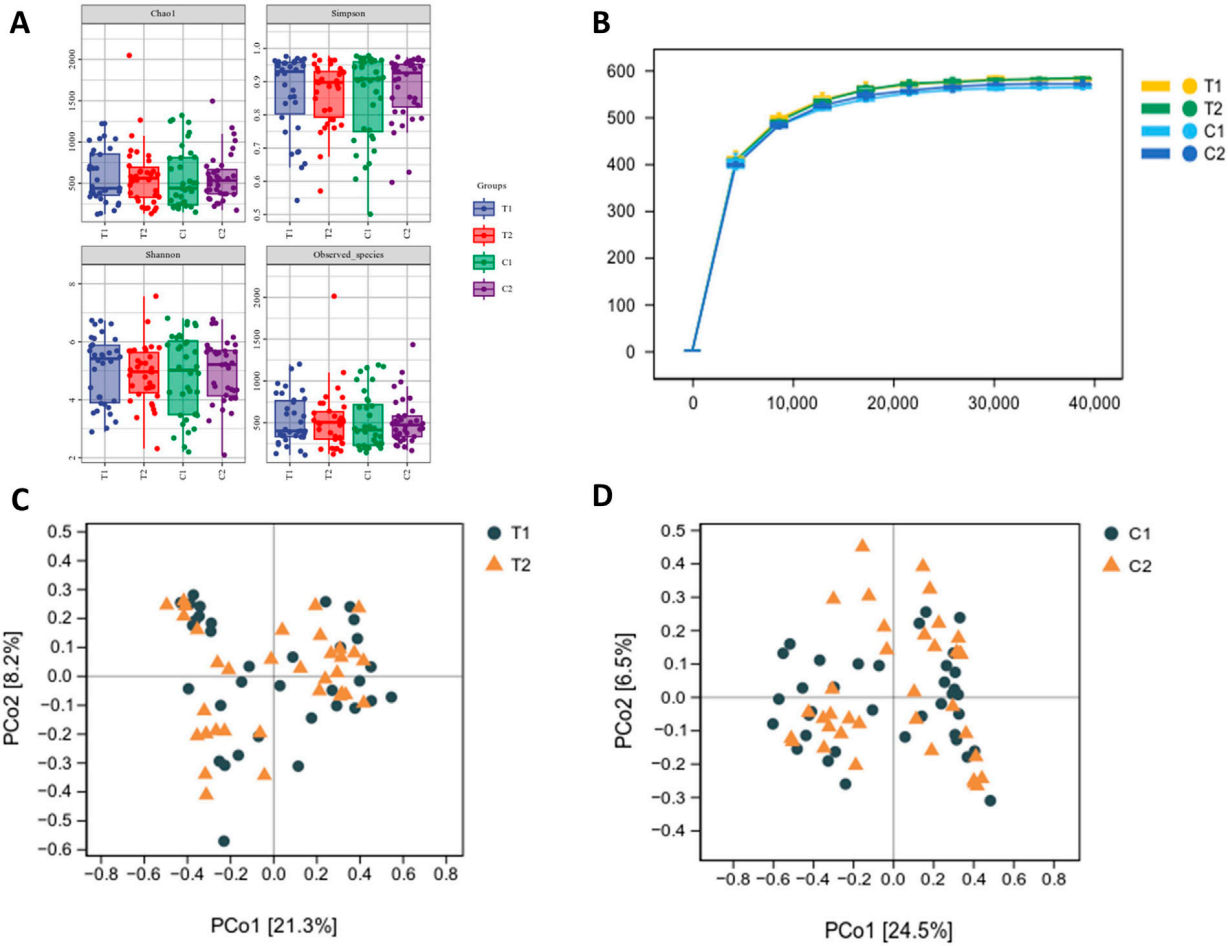
Effects of SKS on gut microbial diversity. **(A)**:α-diversity of the gut microbiome between patientsin the control and SKS group at baseline (C1, T1) and at 4-month follow-up (C2, T2) according to the Chao1, observed species, Shannon, and Simpson diversity indices. **(B)**:The rarefaction analysisbetween the number of samples and the number of ASVs by the Chao1 indices **(C)**:Principal Coordinates Analysis (PCoA) of the gutmicrobiota of patients in the SKS group at baseline (T1) and 8-weekfollow-up (T2). **(D)** Principal Coordinate Analysis (PCoA) of the gut microbiota of patients in the control group at baseline (C1) and at 8-week follow-up (C2).

There were no discernible variations in α diversity as measured by the Chao1, observed species, Shannon, or Simpson diversity indices (Figure 4A), indicating that there were no significant changes in the richness or evenness of the gut microbiota. As the sequencing depth increased, the rarefaction curves began to plateau, suggesting that the current sequencing depth was sufficient for comprehensive microbial community analysis (Figure 4B). The β diversity of the samples was assessed through Principal coordinate analysis (PCoA) using weighted UniFrac metric which reveals no clear separation between the two groups. (Figures 4C,D).

### Associations between the gut microbiota and renal function

To explore the relationships between renal function indicators and the gut microbiota in patients with CKD stages 3–4, Spearman’s correlation analysis was performed, and the specific results are presented in (Figure 3E). The analysis revealed that *Bifidobacterium*, *Blautia*, and Enterococcaceae_*Enterococcus* were negatively correlated with SCrand Cys-C but positively correlated with the eGFR. In contrast, *Parabacteroides*, *Acinetobacter*, *Sutterella*, *Oscillospira*, and *Alistipes* were positively correlated with SCr, BUN, and Cys-C and negatively correlated with the eGFR. These results suggest that SKS may affect renal function in CKD patients by modulating the gut microbiota.

### Adverse reactions

In this study, one patient withdrew due to diarrhea (more than three episodes per day), which significantly impacted daily life and led to voluntary discontinuation. Some participants experienced mild diarrhea, but it was well-tolerated and did not interfere with normal activities. The remaining patients reported no discomfort. Throughout the study, no other adverse events were observed, such as perianal pain, hematochezia (bloody stools), or abdominal pain.

## Discussion

Our randomized controlled trial is the first to investigate the effects of SKS on the gut microbiota and renal function in patients with CKD stages 3–4. Our findings indicate that intervention with SKS may slow the progression of CKD. We observed that SKS induced beneficial alterations in the gut microbiota composition, including an increased abundance of Ruminococcaceae-*Ruminococcus* and Coriobacteriaceae while reducing the abundance of Veillonellaceae. Additionally, we demonstrated a strong association between certain gut microbial taxa and renal function, which may provide a new target for treatment of CKD.

The protocol previously proposed by Mei et al. is based on existing preclinical and clinical evidence supporting the renoprotective effects of Shenkang (Mei et al., 2021). Our results demonstrated a positive impact of SKS on CKD patients after 8 weeks of treatment, which aligns with the findings of previous studies. We demonstrated an increase in the eGFR and a decrease in SCr after 8 weeks of treatment with SKS. The clinical significance of the identified changes in biomarkers, including an increase in eGFR of 3.8 mL/min/1.73 m^2^, a decrease in SCr, and a reduction in Cys-C, is supported by the KDIGO 2024 risk stratification thresholds signifying that “sustained eGFR decline >5 mL/min/1.73  m^2^ year-1 is indicative of accelerated CKD progression”. In contrast, interventions reducing eGFR decline ≥0.75–1 mL/min/year were associated with dialysis initiation delay by 1.5–3 years (Kidney Disease: Improving Global Outcomes CKD Work Group, 2024). Overall, the degree of eGFR improvement demonstrated in our study exceeded the relevant threshold and likely reduced the 5-year risk for kidney failure by a minimum of 3%–5%, the significant threshold for referral for nephrology according to KDIGO. One study indicated that ShenKang treatment protects renal function in diabetic rats by increasing nephrin expression, thereby reducing hyperglycemia-induced kidney damage (Qu et al., 2023). Notably, SKS primarily consists of Rheum palmatum, Astragalus membranaceus, Salvia miltiorrhiza, and Carthamus tinctorius, all of which have been widely reported to exert nephroprotective effects. These components play key roles in anti-inflammatory and antifibrotic effects and improve the eGFR and are widely used in CKD treatment (Zou et al., 2020).

ShenKang has a protective effect on renal function in CKD patients. Emerging studies suggest that this may be related to its influence on the gut microbiota. Rhubarb enema can improve the intestinal barrier, regulate gut microbiota dysbiosis, suppress systemic inflammatory responses, and alleviate renal fibrosis (Ji et al., 2020). Research by Yang Lei et al. showed that rhubarb treatmentimproved intestinal barrier function and promoted the restoration of gut homeostasis (Yang et al., 2022). Astragalus alsocan modulate the gut microbiota in patients, optimize microbial composition, and regulate the immune system and intestinal barrier function (Su et al., 2023).

A key finding of our study is that SKS treatmentinduced changes in the fecal bacterial community composition of CKD patients. The dominant bacterial phyla identified in the colon of our CKD patients were Firmicutes, Proteobacteria, Bacteroidetes, and Actinobacteria. Following SKS treatment, we observed a slight decrease in Bacteroidetes and a slight increase in the Firmicutes/Bacteroidetes (F/B) ratio. Bacteroidetes have been linked to various conditions, especially inflammation (Parker et al., 2020). This decrease could be considered beneficial. Gryp et al. reported that bacterial species involved in phenolic compound production are primarily Bacteroidetes and Firmicutes, suggesting that targeting Bacteroidetes in interventions could lead to decreased inflammation (Gryp et al., 2020). Additionally, SKS significantly increased *Ruminococcus* abundance in our patients. Several studies have shown a significant reduction in Ruminococcaceae abundance in CKD patients, and *Ruminococcus* species are key producers of butyrate (Guirong et al., 2018; Zhang et al., 2020). Butyrate not only maintains intestinal epithelial integrity but also provides energy for epithelial cells and suppresses inflammation by promoting colonic regulatory T-cell differentiation (Guirong et al., 2018). Moreover, SKS treatment significantly reduced the abundance of Veillonellaceae, including *Veillonella*, a facultative pathogen. Liu et al. reported that Veillonellaceae abundance was significantly increased in diabetic patients undergoing hemodialysis (Liu et al., 2020). Veillonellaceae has been linked to obesity, which increases the risk of several diseases, including cancer, atherosclerosis, and diabetes, all of which may accelerate CKD progression (Peters et al., 2018). In contrast, Coriobacteriaceae is linked to good metabolic health, particularly glucose metabolism (Zhuang et al., 2021). Our findings further support the hypothesis that SKS improves metabolic health by modulating the gut microbiota composition. Furthermore, there were no significant differences in diversity between CKD patients, suggesting that bacterial community diversity was not severely disrupted.

LEfSe analysis revealed significant differences in the distributions of many bacterial communities before and after SKS treatment. Planctomycetes, Spirochaetes, Rhodocyclales, Actinomycetaceae, Veillonellaceae, and *Dechloromonas* were significantly enriched before SKS treatment. Among them, Actinomycetaceae is generally considered a harmful intestinal microorganism. Gryp et al. demonstrated that as renal function declines, the abundance of short-chain fatty acid-producing bacteria, such as *Bifidobacterium* and *Streptococcus*, significantly decreases, which aligns with our findings (Gryp et al., 2020). Furthermore, no significant enrichment of *Streptococcus* was observed after SKS treatment. These findings suggest that SKS treatment may improve the gut microbiota composition and serve as a potential mechanism to alleviate dysbiosis in CKD patients.

Previous findings have shown that the composition of the gut microbiota is closely linked to outcomes in CKD patients, including renal function, disease progression, mortality, inflammation, and peritonitis (Voroneanu et al., 2023). Our study confirms the correlation between specific gut microbiota and renal function indicators in CKD patients. Notably, the genera *Bifidobacterium, Blautia*, and Enterococcaceae *Enterococcus* were negatively correlated with the SCr, BUN, and cystatin C levels. In contrast, a positive correlation was found with the eGFR.

*Bifidobacterium* is a short-chain fatty acid-producing bacterium, and a study by Jiang et al. revealed a negative correlation between *Bifidobacterium* and creatinine, as well as between *Bifidobacterium* and BUN, which is consistent with our results (Jiang et al., 2017). Research *in vivo* and *in vitro* demonstrated that *Bifidobacterium* significantly reduces serum concentrations of uremic toxins and has anti-inflammatory and mucosal protective properties, which contribute to improved renal function (Dong et al., 2024; O'Callaghan and van Sinderen, 2016). In recent years, research on *Blautia* as a probiotic has increased, and *Blautia* is known for its ability to produce SCFAs and its ability to improve metabolism (Liu et al., 2021; Hosomi et al., 2022). Additionally, we identified five bacterial genera that were positively correlated with clinical renal function indicators. Studies have shown that the abundances of *Parabacteroides, Acinetobacter (* Al-Obaide et al., 2017), *Sutterella,* (Du et al., 2021), *Oscillospira* (Zhang et al., 2020) and *Alistipes* (Wang et al., 2020) are increased in patients with CKD. Among them, *Oscillospira* is associated with mortality (Lin et al., 2021) and inflammation (Margiotta et al., 2020). *Parabacteroides* encodes the urease gene, which promotes inflammatory uremic toxins (Wong et al., 2014). These findings suggest that these bacteria could serve as noninvasive biomarkers for the early diagnosis of CKD, suggesting new research directions for the early detection of CKD in the future.

With respect to the safety of SKS, previous small-sample randomized controlled trials have not reported significant adverse reactions. In our study, no significant adverse events occurred. Although SKS may lead to increased bowel frequency and a stronger urge to defecate, all participants were able to tolerate these reactions. However, these side effects may have somewhat reduced adherence to the treatment protocol. In uremic patients, changes in the gut microbiota composition are related to reduce fiber intake, which can impair gut motility and increase the incidence of constipation (Yasuda et al., 2002). SKS was found to alleviate constipation symptoms to some extent.

Despite our findings, there are a few limitations to acknowledge. First, we did not quantify the participants’ dietary intake, although their dietary habits were generally stable, and all participants came from neighboring towns around the hospital and shared similar dietary patterns. This may reduce the reproducibility of the experiment. Second, this was a single-center, small-scale exploratory study, and the sample size limited the statistical power to detect significant effects of SKS on certain secondary outcomes or indicators. Moreover, this study was conducted over a relatively short duration, limiting our ability to evaluate the long-term effects of SKS on the gut microbiota and renal function. Additionally, the current study design did not incorporate a crossover washout phase, which could help differentiate the specific effects of SKS from potential placebo effects or temporal variations. Moreover, this study only observe 8 weeks. Future larger long-term studies with a crossover washout design are needed to assess the effects of SKS on the gut microbiota and the progression of CKD more comprehensively. Lastly, 16srRNA sequencing has several limitations, including limited resolution, lack of functional information, and measurement of relative rather than absolute abundance. Future studies should integrate metagenomic sequencing or metabolomics for more comprehensive investigations.

## Conclusion

The current study revealed positive effects of SKS in the treatment of CKD stage 3–4 patients, potentially slowing the progression of CKD. SKS also led to beneficial changes in the composition of microbiomes. Notably, the correlation between changes in renal function and specific microbes suggests that targeting the microbiota may serve as a potential therapeutic approach for CKD patients. However, further experiments are needed to validate these findings.

## Data Availability

The datasets presented in this study can be found in online repositories. The names of the repository/repositories and accession number(s) can be found below: NCBI BioProject (https://www.ncbi.nlm.nih.gov/bioproject/), accession number PRJNA1292718.

## Abbreviations

CKD, Chronic kidney disease; SKS, Shenkang Suppository; LDA, Linear Discriminant Analysis; ESKD, End-Stage Kidney Disease; SCFAs, short-Chain Fatty Acids; CVD, Cardiovascular Disease; TCM, Traditional Chinese Medicine; BMI, Body Mass Index; SBP, Systolic Blood Pressure; DBP, Diastolic Blood Pressure; UA, Uric Acid; BUN, Blood Urea Nitrogen; SCr, Serum Creatinine; eGFR, estimated Glomerular Filtration Rate; Cys-C, Cystatin C; K, Potassium; ALB, Serum Albumin; TC, Total Cholesterol; GLU, Glucose; Hgb, Hemoglobin; P, Phosphate; ASVs, Amplicon Sequence Variants; F/B, Firmicutes/Bacteroidetes; LEfSe, LDA effect size; PCoA, Principal Coordinate Analysis.
